# A silk fibroin/decellularized extract of Wharton’s jelly hydrogel intended for cartilage tissue engineering

**DOI:** 10.1007/s40204-019-0108-7

**Published:** 2019-01-31

**Authors:** Arefeh Basiri, Mehdi Farokhi, Mahmoud Azami, Somayeh Ebrahimi-Barough, Abdolreza Mohamadnia, Morteza Rashtbar, Elham Hasanzadeh, Narges Mahmoodi, Mohamadreza Baghaban Eslaminejad, Jafar Ai

**Affiliations:** 1 Department of Tissue Engineering and Applied Cell Sciences, School of Advanced Technologies in Medicine, Tehran University of Medical Sciences, Tehran, Iran; 2 National Cell Bank of Iran, Pasteur Institute of Iran, Tehran, Iran; 3 Department of Biotechnology, School of Advanced Technologies in Medicine, Shahid Beheshti University of Medical Sciences, Tehran, Iran; 4 Sina Trauma and Surgery Reasearch Center, Tehran University of Medical Sciences, Tehran, Iran; 5 Department of Stem Cells and Developmental Biology, Cell Science Research Center, Royan Institute for Stem Cell Biology and Technology, ACECR, Tehran, Iran

**Keywords:** Decellularization, Wharton’s jelly, Silk fibroin, Hydrogel, Cartilage tissue engineering

## Abstract

A hybrid hydrogel was obtained from decellularized extract from Wharton’s jelly (DEWJ) and silk fibroin (SF) and characterized for cartilage tissue engineering. Wharton’s jelly was used due to its similarity with articular cartilage in extracellular matrix composition. Also, silk fibroin has good mechanical properties which make this construct appropriate for cartilage repair. Decellularization of Wharton’s jelly was verified by DAPI staining, DNA quantification, and PCR analysis. Then, the biochemical composition of DEWJ was determined by ELISA kits for total proteins, collagens, sulfated glycosaminoglycans (sGAG), and transforming growth factor β1 (TGF-β1). After fabricating pure SF and SF/DEWJ hybrid hydrogels, their physical and mechanical properties were characterized by FESEM, Fourier-transform infrared spectroscopy (FTIR) and rheological assays (amplitude and frequency sweeps). Furthermore, cell viability and proliferation were assessed by MTT assay. The results have shown that DEWJ in hybrid hydrogels enhances mechanical properties of the construct relative to pure SF hydrogels. Also, this extract at its 40% concentration in culture media and 20% or 40% concentrations in SF/DEWJ hybrid hydrogels significantly increases population of the cells compared to control and pure SF hydrogel after 7 days. In conclusion, this study proposes the potential of SF/DEWJ hybrid hydrogels for cartilage tissue engineering applications.

## Introduction

Tissue engineering is a promising approach for repairing cartilage injuries by culturing cells under controlled conditions within a suitable three-dimensional scaffold. Different biomaterials have been designed to support growth and differentiation of cultured cells to form functional tissues by providing appropriate physical and chemical milieu. Mesenchymal stem cells (MSCs) are utilized as a common cell source in tissue engineering (Hao et al. 2013; Sundaram et al. 2017). The important characteristics of these cells such as survival, self-renewal, and differentiation highly depend on their surrounding extracellular matrix (ECM) (Hao et al. 2013; Kusuma et al. 2017). Therefore, one of the major challenges in this field is to provide a suitable biomaterial which is able to mimic the native niche of cells properly (Herrero-Mendez et al. 2017). There are various biodegradable materials including synthetic and natural ones for scaffold fabrication. Natural materials induce more cell adhesion, proliferation, and matrix secretion than synthetic ones (Zhao et al. 2018). ECM is a natural material which is usually obtained from native tissues or in vitro matrices secreted by cultured cells (Benders et al. 2013; Cha et al. 2013; Yang et al. 2009).

The ECM derived from different tissues is a rich reservoir of bioactive molecules regulating the proliferation, differentiation, and migration of cells (Swinehart and Badylak 2016; Taylor-Weiner et al. 2013; Wolf and Friedl 2011). Due to these appropriate characteristics, there is a significant interest for using tissue decellularization to provide ECM-based scaffolds (Choi et al. 2012; Costa et al. 2007; Hellström et al. 2014; Kiyotake et al. 2016; Law et al. 2016).

Wharton’s jelly (WJ) is a mucous connective tissue that surrounds the umbilical cord vessels and is covered by a layer of simple amniotic epithelium (Converse et al. 2017; Sobolewski et al. 2005). ECM components of WJ are similar to those of articular cartilage (Xiao et al. 2017). Collagen is a basic part of WJ-ECM which organizes above 50% of the defatted dry weight of tissue. Furthermore, WJ has large amounts of glycosaminoglycans (GAGs), especially hyaluronic acid (HA) which forms about 70% of total GAG content (Sobolewski et al. 1997). The high percentage of hyaluronic acid (HA) makes this tissue strongly hydrated, viscous, and suitable as a natural hydrogel-type biomaterial (Ferguson and Dodson 2009). Additionally, WJ is a rich source of cytokines such as insulin-like growth factor 1 (IGF-1), platelet derived growth factor (PDGF), basic fibroblast growth factor (bFGF), and transforming growth factor-β (TGF-β) which are associated with ECM proteins and can help in controlling cellular processes such as proliferation and differentiation (Jadalannagari et al. 2017; Sobolewski et al. 2005). The possibility of obtaining umbilical cord as a sub-product with a wide range of sources without ethical concerns enhances WJ merits for biomedical applications (Herrero-Mendez et al. 2016).

Silk fibroin is another natural biomaterial which is extracted from the cocoons of a silkworm and has exclusive properties in biological applications and scaffold synthesis (Zubir and Pushpanathan 2016). It has low immunogenicity and inflammatory response as well as suitable biocompatibility and biodegradability, unique biochemical and mechanical properties (Bhardwaj et al. 2011). Moreover, it can be used to fabricate various types of scaffolds with different methods especially in the case of hydrogels (Kapoor and Kundu 2016; Li et al. 2013; Uebersax et al. 2008; Wang et al. 2008; Yucel et al. 2009). In a previous report, a lyophilized SF hydrogel produced by sonication method followed by freeze drying was introduced as a good delivery matrix with the ability of long-term sustained release. In addition, the possibility of adding drug or supplement after sonication removes shear stress effect on the structure and bioactivity of the drug or supplement in the construct (Guziewicz et al. 2011).

Because SF is a known biomaterial in a broad range of tissue engineering applications, it can be used as the base material in fabricating a cartilage tissue-engineered construct which can carry and deliver supplements to stem cells to induce their proliferation and differentiation.

In this study, the DEWJ was obtained from a human source by a mild procedure. After characterization of the main components of DEWJ which are similar to those of cartilage ECM such as total protein, soluble collagen, insoluble collagen, total collagen, TGF-β1, and sGAG, the best extract and its concentrations in culture media and SF-based hydrogels for viability of human endometrial stem cells (hEnSCs) were determined. Some advantages of hEnSCs over other sources of stem cells including a greater ease of supply, the long availability during a woman’s life cycle, obtaining stem cells from a waste tissue without critical ethical issues (Verdi et al. 2014) and maintained proliferative potency in elderly women (Ebrahimi-Barough et al. 2013) lead to choose this type of stem cells for viability and proliferation assay. Furthermore, the capacity of differentiation into mesenchymal tissue cells such as chondrocyte-like cells, reported by Wolf et al. (2007) and Sekine et al. (2013), makes hEnSCs a promising source for the future of cartilage tissue engineering. In addition, the blend of DEWJ and silk fibroin has not been studied so far. In previous studies an extract of Wharton’s jelly has been used as a coating material (Dan et al. 2017; Hao et al. 2013) and has not been blended with a material to form the whole structure of the scaffold which influences on physicochemical properties of the scaffold along with improving cell attachment and proliferation. Therefore, in the present work, it was tried to enhance the properties of SF-based hydrogels by adding DEWJ benefits to develop a new cartilage tissue-engineered construct.

## Materials and methods

### Decellularization of Wharton’s jelly

Umbilical cords of full-term pregnancy were obtained from consenting mothers after normal vaginal delivery at Valiasr Hospital (Tehran, Iran), and were forthwith placed in a transport solution including HANKS media supplemented with 1% penicillin/streptomycin (Sigma-Aldrich, USA), and amphotericin 0.25 mg mL^−1^ (Sigma-Aldrich, USA). Then, the samples were transferred to the lab on ice. In a laminar flow safety cabinet, the umbilical cords were rinsed with phosphate-buffered saline (PBS, Sigma-Aldrich, USA) containing 2% antibiotic/antimycotic as a washing solution. Next, they were cut to 4–5 cm pieces and the surrounding membranes and vascular structures were removed. The jelly was scraped by a scalpel and completely sliced. The obtained jelly (approximately 5 mL from every sample) was then placed in a 50 mL conical tube with deionized water at 1:3 ratio. Then, the immediate or overnight extracts were centrifuged at 4 °C to remove the cells and cord debris. The supernatant as DEWJ was collected and frozen at − 80 °C until use.

### Optimization of DEWJ

To determine a better centrifuge round and time for decellularization of the extracts, we chose three groups including (1) 5000 rpm for 15 min, (2) 5000 rpm for 30 min, (3) 10,000 rpm for 15 min. Then, DNA quantification test was performed on all the three groups (*n* = 3). The best group was defined and used for later experiments.

It was hypothesized that the time at which WJ is exposed to deionized water may affect the concentration of the materials extracted by the method proposed in this study. Thus, centrifugation was performed at two time points: (1) immediately after adding deionized water (immediate extract), (2) overnight after adding deionized water (overnight extract). Then, biochemical compositions were evaluated for native WJ, immediate extract, and overnight extract.

### Verification of WJ decellularization

#### Quality assessment of DEWJ

To verify cell removal in the extracts after different centrifugation treatments based on round and time, native WJ and its decellularized extracts were stained with DAPI (Sigma-Aldrich, USA) and observed under a fluorescent microscope (Olympus BX51, Japan).

#### DNA quantification

To determine the total DNA content of native WJ and its decellularized extracts, 1 mg of each lyophilized sample was digested using 200 µL proteinase K (Invitrogen, USA) for 2–3 h. Total genomic DNA of native WJ and its decellularized extracts (*n* = 3) were isolated by PrimePrep Genomic DNA Isolation Kit (Genet Bio, South Korea) following the manufacturer’s instructions. The total amount of DNA was quantified by spectrophotometry (Nano Drop ND-0910, Thermo Scientific, USA). The ratio of optical densities of 260/280 nm indicated the purity and yield of nucleic acids, and absorbance in 260 nm showed the DNA quantity. The analysis was performed on three different donor samples of WJ.

#### Polymerase chain reaction (PCR)

The following primers were used to amplify β-globin housekeeping gene by PCR: forward primer 5′-ACACAACTGTGTTCACTAGC-3′ and reverse primer 5′-CAACTTCATCCACGTTCACC-3′. The estimated length of PCR product was 110 bp and the total volume of PCR mixture was 25 µL which contained 2 µL of the forward and reverse primers, 12 µL of amplicon red master mix (Amplicon, Denmark), 1 mL of self-stain (SinaClon BioScience Co, Iran), 5 µL of DNA, 5 µL of DNAase/RNAase-free distilled water using the following thermal cycles: an initial denaturation at 94 °C for 4 min, 30 cycles of 94 °C for 30 s, 55 °C for 30 s, and 72 °C for 30 s and a final extension at 72 °C for 5 min. PCR products were analyzed by electrophoresis on a 2% agarose gel stained with loading dye.

### Biochemical analysis

To study the components of native WJ and its decellularized extracts, biochemical assays of total protein, collagen (soluble and insoluble), sGAG, and TGF-β1 were performed as described in the following sections.

#### Protein quantification

Total WJ proteins were examined by bicinchoninic acid (BCA) method using the Pierce BCA Protein assay kit (Thermoscientific, USA) and according to the manufacturer’s instructions for microplate procedure. Briefly, 25 μL of each standard or sample replicate was poured into a microplate well of 96-well plates, and then 200 μL of the BCA Working Reagent (WR) was added to each well and mixed thoroughly on a plate shaker for 30 s following the incubation of the plate at 37 °C. After 30 min, the plate was cooled to room temperature, and the colorimetric reactions were measured at 562 nm using amultiplate reader (H4, BIO-TEK Instruments Inc., USA). The analysis was performed on three different donor samples of native WJ and its decellularized extracts. The total protein amount of each sample was estimated using a standard curve for bovine serum albumin (BSA).

#### Collagen quantification

Soluble and insoluble collagen contents of the samples were extracted and quantified using Sircol soluble and insoluble collagen assay kits (Biocolor, United Kingdom) based on the manufacturer’s instructions. Briefly, to extract acid–pepsin-soluble collagen, the specimens were digested with 0.5 M acetic acid containing 1 mg mL^−1^ pepsin (P7012; Sigma-Aldrich, USA) overnight at 4 °C. To extract insoluble collagen, the specimens were placed in fragmentation reagent in a 60 °C water bath for 3 h. The soluble and insoluble collagens were incubated with 1 mL Sircol dye reagent at room temperature for 30 min. The collagen–dye complex was precipitated by centrifugation at 10,000 rpm for 10 min and the supernatant was removed. The pellets were dissolved in 1 mL alkaline reagent and the relative absorbance was recorded at 555 nm for soluble collagen and at 550 nm for insoluble collagen in a 96-well plate using a multiplate reader (H4, BIO-TEK Instruments Inc., USA). Total collagen amount was calculated by summing the amounts of the two collagen types.

#### Sulfated GAG quantification

The sGAG content in the DEWJ was measured using a Blyscan sulfated GAG assay kit (Biocolor, UK) according to the manufacturer’s instructions. Briefly, to extract sGAG, the samples were digested with a papain solution (with a concentration of 125 mg mL^−1^ papain in GAG buffer) (Sigma-Aldrich, USA) and placed in a water bath at 60 °C overnight. The suspension was centrifuged at 10,000*g* for 10 min. About 100 µL of the supernatant containing sGAG was mixed with 1 mL Blyscan dye and shaken for 30 min. The precipitate was collected by centrifugation at 12,000 rpm for 10 min and then dissolved in 1 mL of dissociation reagent. The absorbance was measured in a 96-well plate at 656 nm using a multiplate reader (H4, BIO-TEK Instruments Inc., USA).

#### TGF-β1 quantification

TGF-β1 concentrations were analyzed using an enzyme-linked immunosorbent assay kit (Human TGF Beta 1 PicoKine™ ELISA Kit, USA) and recombinant human active TGF-β1 as standards according to the manufacturer’s instructions. Briefly, each sample and standard were added to each well of the 96-well plate and incubated for 90 min. Then, biotinylated antibodies were added and incubated for 60 min. After washing the plate three times with 0.01 M TBS, ABC working solution was added and incubated for 30 min. Afterward, the plate was washed five times with 0.01 M TBS and TMB color developing agent was added and incubated in dark for 15–20 min. Finally, TMB stop solution was added and the absorbance was measured at 450 nm using a multiplate reader (H4, BIO-TEK Instruments Inc., USA). All the incubations were performed at 37 °C.

### Human endometrial stem cell culture

hEnSCs were prepared from Iranian Biological Resource Center (IBRC C10128) and cultured in DMEM-F12 supplemented with 10% fetal bovine serum (FBS) and 1% penicillin/streptomycin. Passage 3 of these cells was used for MTT assay.

### Viability and proliferation assay for different concentrations of DEWJ in culture media

To evaluate the proliferation of different concentrations of DEWJ, 3-(4,5-dimethylthiazol-2-yl)-2,5-diphenyl tetrazolium bromide (MTT; Sigma, USA) assay was performed in a 96-well cell culture plate. We chose the overnight extract for this assay. Next, hEnSCs were seeded at the density of 5 × 10^3^ cell per well. About 200 µL of a serum-free culture medium (DMEM-F12) supplemented with different percentages of DEWJ (2.5, 5, 10, 20, 30, 40, and 50%) was added to different wells to determine its influence on the proliferation of hEn-SCs (*n* = 3). Then, the plates were placed in the incubator with 37 °C and 5% CO_2_. After the definite time points (1, 3, and 7 days), the culture medium was removed and 100 µL new serum-free culture medium and 10 µL MTT solution (0.5 mg mL^−1^) were added to each well. After 3 h of incubation at 37 °C, MTT solution was removed and 100 µL dimethyl sulfoxide (DMSO) (Merck, Germany) was added. Then, 100 µL of the supernatant obtained from each well was transported to the well of a new 96-well cell culture plate and the absorbance of the formazan product was measured at 570 nm using a multiplate reader (H4, BIO-TEK Instruments Inc., USA).

### Preparation of SF extract

SF was obtained by following previous reported protocols (Sobolewski et al. 1997, 2005). Briefly, the cocoons of mulberry *Bombyx mori* silkworm were boiled in 0.2 M of Na_2_CO_3_ solution followed by washing in distilled water and drying at room temperature to produce degummed fibers. After dissolving these fibers in 9.3 M of LiBr solution and dialyzing against deionized water, the obtained SF solution with final concentration of 4% (w/v) in water was preserved at 4 °C until use.

### Preparing SF-based hydrogels

To fabricate hydrogels, SF was used as a base material and DEWJ was added as a supplement before the gelation of SF. For induction of gelation, SF solution 4% (w/v) was sonicated at 40% amplitude for about 15 s on an ice bath. Then, DEWJ was mixed with the sonicated SF solution at 20% and 40% concentrations (v/v). All the hydrogels including pure SF, SF/20% DEWJ and SF/40% DEWJ were incubated at 37 °C to complete the gelation process. Then, the hydrogels were frozen overnight at − 20 °C and another overnight at − 80 °C followed by lyophilization in a freeze drier for 48 h to produce the lyophilized hydrogels.

### Fourier transform infrared spectroscopy (FTIR) analysis

Infrared spectra of the emission of lyophilized hydrogels were obtained in the range of 400-4000 cm^−1^ with a resolution of 4 cm^−1^ using an FTIR spectrometer (Thermo Nicolet, Nexus 670). The spectra of the samples were measured at room temperature and the data were analyzed using OriginPro 2017 software.

### Rheological study

Oscillatory rheological characterization of hydrogels including amplitude sweep and frequency sweep was evaluated using Physica MCR 502 (Anton Paar). For all the experiments, the hydrogels with a diameter of 30 mm were used (the parameters of the rheometer are mentioned in Table 1). Amplitude sweep test was performed for the determination of linear-viscoelastic regime (LVE) range which was used in the frequency sweep test for choosing the constant strain. Furthermore, storage modulus (*G*′) and loss modulus (*G*′′) were obtained by the frequency sweep test to predict the viscoelastic behavior of the hydrogels.

**Table 1:** Parameters of the rheometer

Parameter	Strain sweep	Frequency sweep
Temperature (°C)	25	25
Parallel plate size (mm)	25	25
Gap size (mm)	1	1
Frequency (Hz)	Constant: 0.1	0.01–100
Strain (%)	0.01–100	Constant: 0.1

### Preparing hydrogels for cell culture

Pure and hybrid hydrogels were sterilized by ethanol followed by UV light treatment of each side of the hydrogels. The samples were washed 3 times with PBS and placed in the culture media for 3 h. Then, hEnSCs were seeded at a density of 5 × 10^4^ cells per sample and cultured in DMEM supplemented with 10% FBS and 1% penicillin/streptomycin at 37 °C and 5% CO_2_.

### Evaluation of hydrogel morphology

The hybrid and pure hydrogels were studied by a cold field emission scanning electron microscope (FESEM) (S-4160, Hitachi, Japan) to evaluate morphology and pore properties of hydrogels. The pore size of each hydrogel was reported as the average size of 30 random pores measured using Image J software (NIH, USA).

### Viability and proliferation assay for different hydrogels

To evaluate the effect of different hydrogels including pure SF, SF/20% DEWJ and SF/40% DEWJ on viability and proliferation of hEnSCs, MTT assay was performed after 1, 3, and 7 days according to the above-mentioned protocol.

### Statistical analysis

To calculate statistically significant differences between groups, one-way analysis of variance (ANOVA) was performed followed by a Tukey’s multiple comparison post-test (as appropriate). The results of all experiments were presented as mean ± standard deviation (SD) (*n* = 3) and *P* < 0.05 was accepted as statistically significant.

## Results

### Preparation of DEWJ

**Fig. 1:**
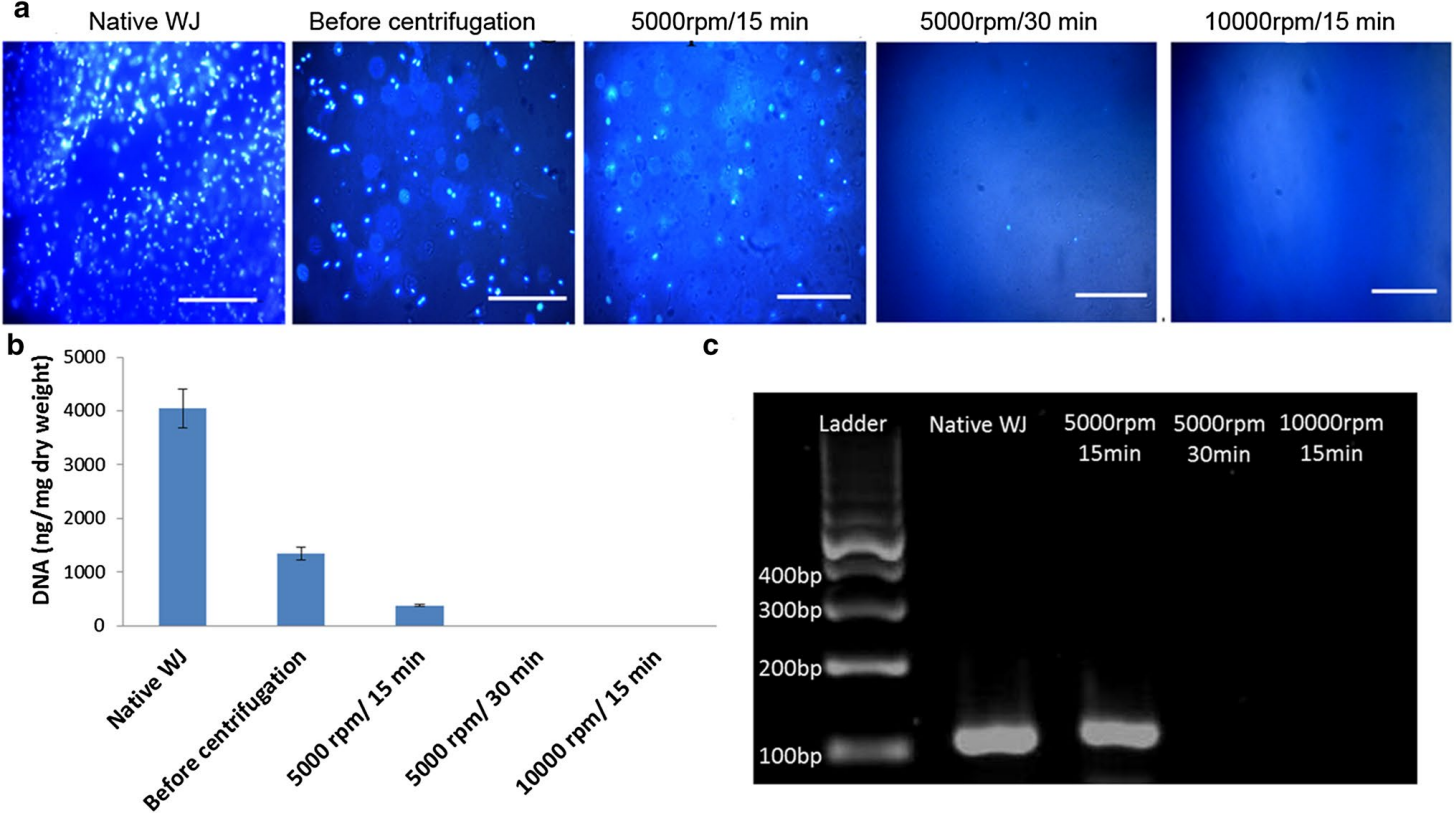
Verification of WJ decellularization by **a** DAPI staining (scale bar = 100 µm), **b** DNA quantification assay (data are shown as mean ± standard deviation, *n* = 3), and **c** agarose gel of the PCR products for native WJ, extract before centrifugation, extracts after centrifugation at 5000 rpm/15 min, 5000 rpm/30 min, and 10,000 rpm/15 min. *DEWJ* dcellularized extract from Wharton’s jelly, *WJ* Wharton’s jelly

The rich WJ-ECM was extracted to obtain a new biomimetic material for tissue engineering applications. For this purpose, WJ was isolated from human umbilical cords and purified after removing the extra tissues and then DEWJ was prepared. DAPI staining of WJ extracts did not reveal any DNA at 5000 rpm/30 min and 10,000 rpm/15 min groups unlike native WJ, extract before centrifuge, and 5000 rpm/15 min groups (Fig. 1a). Furthermore, the DNA quantification and PCR assay also showed the total removal of DNA content at 5000 rpm/30 min and 10,000 rpm/15 min groups and the presence of DNA in the native WJ (4.05 ± 0.35 μg mg^−1^), extract before centrifuge (1.35 ± 0.11 μg mg^−1^), and 5000 rpm/15 min (0.37 ± 0.02 μg mg^−1^) groups (Fig. 1b, c). Although the amount of DNA at 5000 rpm/15 min group was significantly less than those of the other two groups (native WJ and extract before centrifuge), it is still more than the acceptable quantity for decellularized tissues (< 50 ng mg^−1^ dry weight). These results indicate the successful removal of nuclear components from the WJ extracts using centrifugation at 5000 rpm/30 min and 10,000 rpm/15 min groups.

### Biochemical assay of DEWJ

**Fig. 2:**
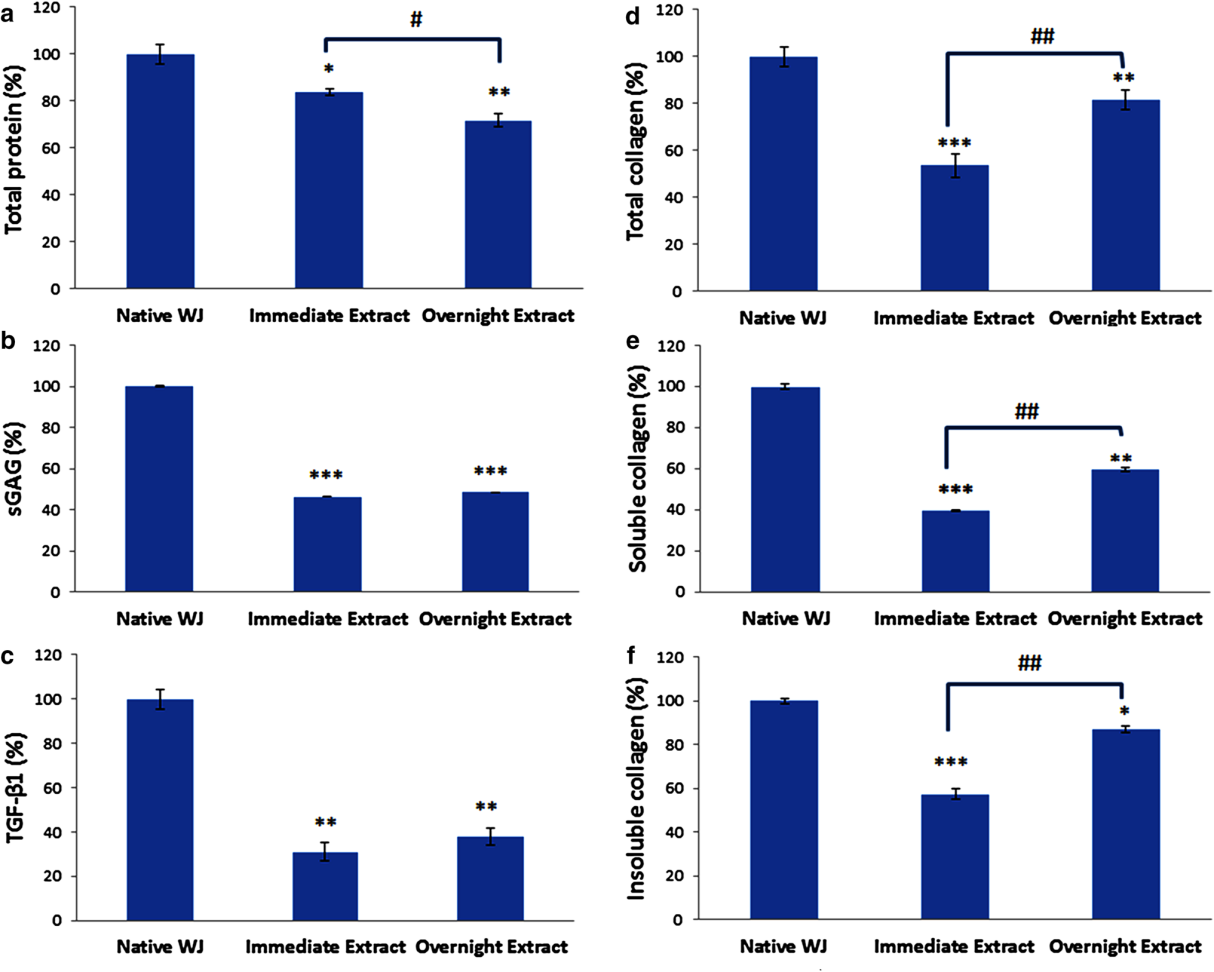
Biochemical analysis of native WJ, immediate extract, and overnight extract in terms of **a** total protein, **b** sGAG, **c** TGF-β1, **d** total collagen, **e** soluble collagen, **f** insoluble collagen. Data are shown as mean ± standard deviation (*n* = 3). * and ^#^ symbols, respectively, indicate comparison with native WJ and immediate extract groups (*P < 0.05, **P < 0.01, ***P < 0.001, ^#^*P* < 0.05, and ^##^*P* < 0.01). *WJ* Wharton’s jelly, *sGAG* sulfated glycoseaminoglycan, *TGF-β1* transforming growth factor-beta 1

To analyze WJ-ECM components (total protein, total collagen, sGAG, and TGF-β1), biochemical assays were performed for native WJ and its decellularized extracts (immediate and overnight) as demonstrated in Fig. 2. In the case of total protein, there was a significant decrease in the overnight extract compared with the immediate extract (12%, *n* = 3, *P* < 0.05; Fig. 2a). Moreover, the amounts of sGAG and TGF-β1 had a minimal increase in the overnight extract (2.28% and 6.78% respectively; Fig. 2b, c). The amounts of soluble collagen, insoluble collagen, and total collagen significantly increased in the overnight extract compared with the immediate extract (20.25%, 29.81%, 27.89%, respectively, *n* = 3, *P* < 0.01; Fig. 2d–f). Therefore, our results indicated that the overnight extract contained more collagen, sGAG, and TGF-β1 than the immediate extract.

### Cell viability and proliferation influenced by different concentrations of DEWJ in culture media

**Fig. 3:**
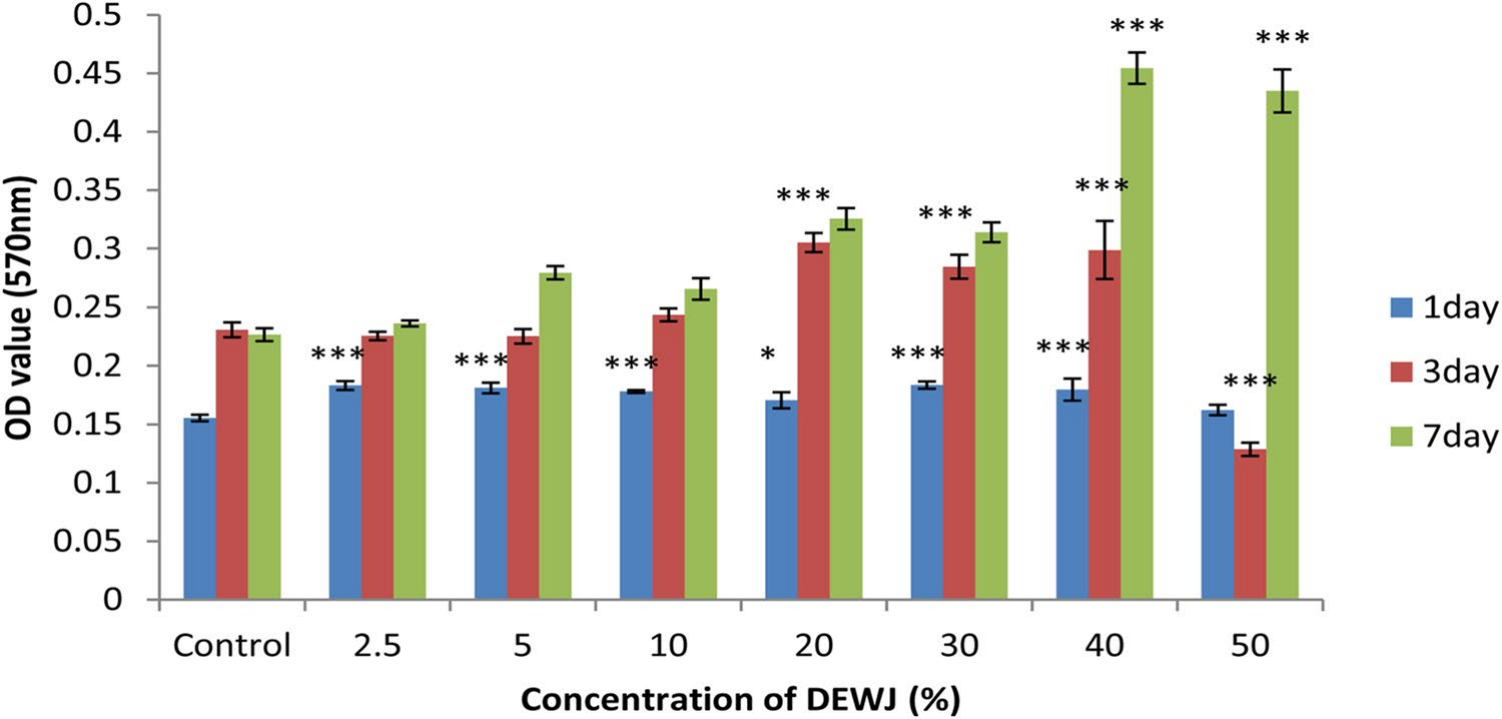
MTT results for different concentrations of DEWJ using hEnSCs. Data are shown as mean ± standard deviation (*n* = 3). * symbol indicates comparison with control group (0%) at same time points (*P < 0.05 and ***P < 0.001). *DEWJ* dcellularized extract from Wharton’s jelly, *hEnSCs* human endometrial mesenchymal stem cells

To evaluate the potential of different concentrations of DEWJ as appropriate inducers for stem cell proliferation, hEnSCs were cultured in serum-free culture media supplemented with 2.5, 5, 10, 20, 30, 40, and 50% of DEWJ and were assessed using the MTT quantitative assay after 1, 3, and 7 days. As shown in Fig. 3, a significant increase in cell population was detected for all concentrations except 50% on day 1, for 20, 30, and 40% concentrations on day 3 and for 40% and 50% concentrations on day 7 relative to the control group (0%). Based on these results, 40% of DEWJ can significantly protect the continuous viability and proliferation of the hEnSC compared with other concentrations and control group during the whole period of the study (*P* < 0.001).

### Fourier transform infrared spectroscopy (FTIR) analysis

**Fig. 4:**
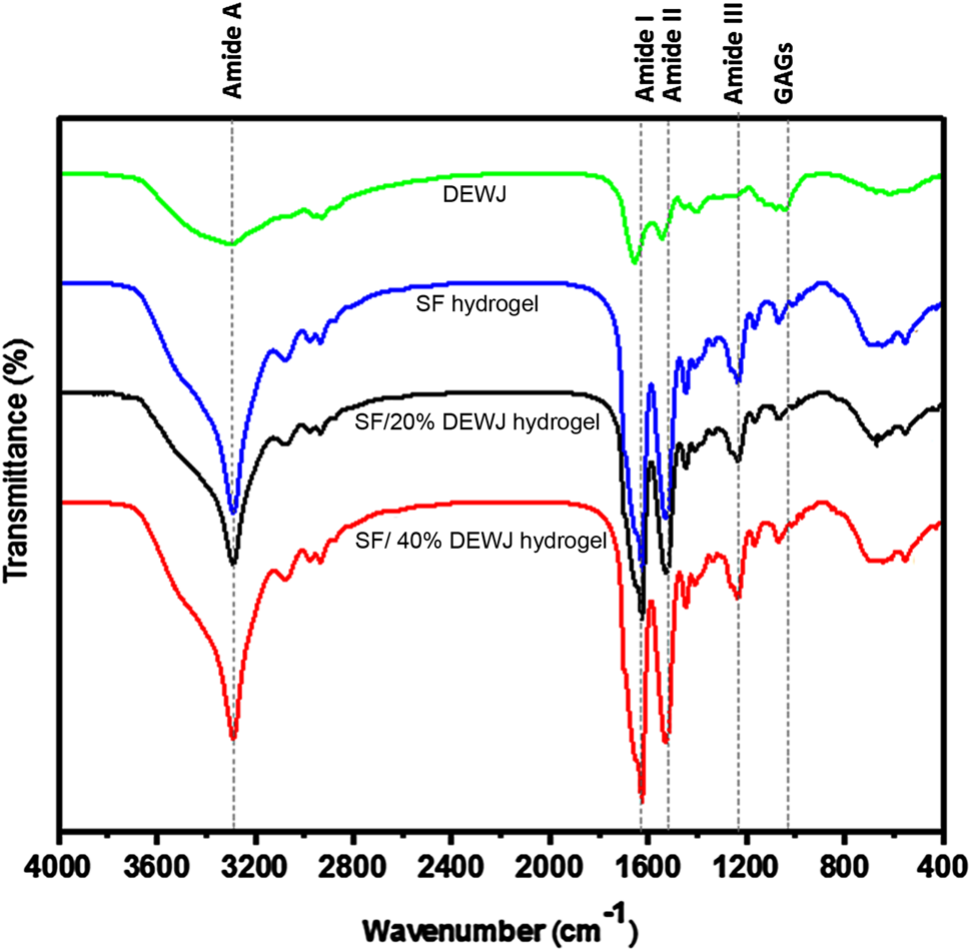
FTIR spectra of DEWJ, SF, SF/20% DEWJ, and SF/40% DEWJ lyophilized hydrogels. Dotted lines demonstrate the center of amide A, amide I, amide II, amide III and GAGs peaks

FTIR spectra revealed the relatively similar structural compositions of pure and hybrid hydrogels and DEWJ. Typical peaks centered at 3295 cm^−1^ (amide A), 1631 cm^−1^ (amide I), 1537 cm^−1^ (amide II), and 1241 cm^−1^ (amide III) verified the protein nature of the samples (Taylor-Weiner et al. 2013). In the case of DEWJ, the peaks mentioned above were weaker with a slight shift for amide I and amide II. Also, another peak centered at 1111 cm^−1^ revealed the presence of polysaccharides (GAGs) in the sample. Sharp peaks belonging to amide I and amide II in all hydrogels represent the β-sheet structure which is increased during the gelation process (Sekine et al. 2013) (Fig. 4).

### Rheological characterization

**Fig. 5:**
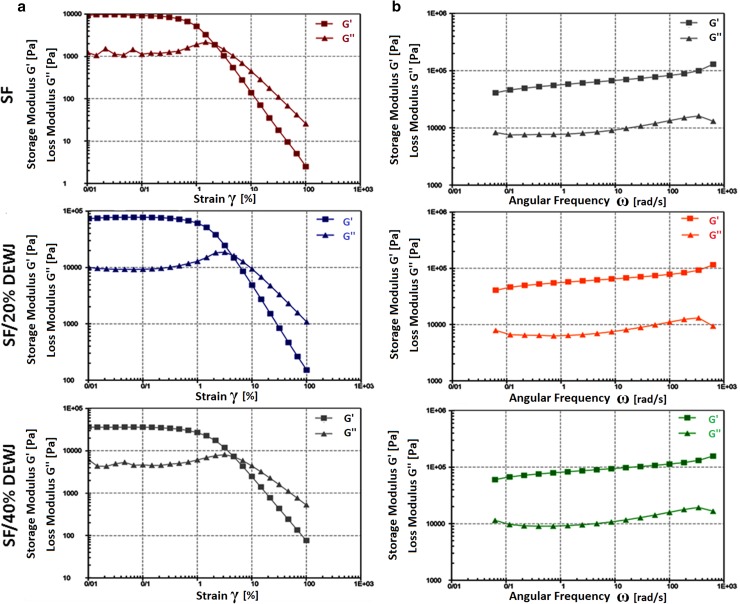
Rheological characterization including **a** amplitude sweeps and **b** frequency sweeps demonstrates storage modulus (*G*′) and loss modulus (*G*′′) of SF, SF/20% DEWJ and SF/40% DEWJ hydrogels

During the amplitude sweep test, elastic and viscous behaviors of hydrogels were evaluated by storage modulus (*G*′) and loss modulus (*G*′′), respectively (Fig. 5a). At the beginning of the chart, there was a linear-viscoelastic (LVE) region where *G*′ and *G*′′ maintained constant values as strain values increased. In the LVE region of all hydrogels, *G*′ indicated higher values than *G*′′. Afterward, *G*′ and *G*′′ started to decrease and increase, respectively, intersecting each other. The intersection point was about 2.5% for pure SF hydrogel and about 5% for SF/20% DEWJ and SF/40% DEWJ hybrid hydrogels. Then, the frequency sweep test was carried out in a constant strain chosen based on LVE region of the previous test. During the test, *G*′ indicated higher values than Gʺ and both moduli had a slight increase in all hydrogels (Fig. 5 b).

### Evaluation of hydrogel morphology

**Fig. 6:**
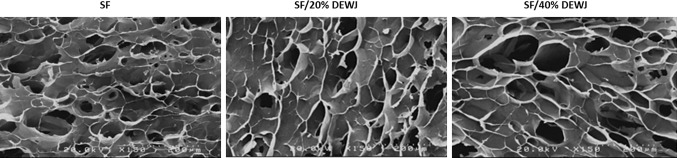
SEM images of SF, SF/20% DEWJ, SF/40% DEWJ lyophilized hydrogels (scale bar = 200 μm)

The microstructure of the fabricated lyophilized hydrogels which was investigated by FESEM indicated heterogeneous pores with interconnectivity in all types of hydrogels (Fig. 6). The mean pore sizes for SF, SF/20% DEWJ, and SF/40% DEWJ hydrogels were estimated as: 52.489** ± **14.111, 98.012** ± **21.743, and 123.042±39.281, respectively. The hybrid hydrogels showed significantly higher values compared with that of pure SF hydrogel.

### Cell viability and proliferation influenced by different hydrogels

**Fig. 7:**
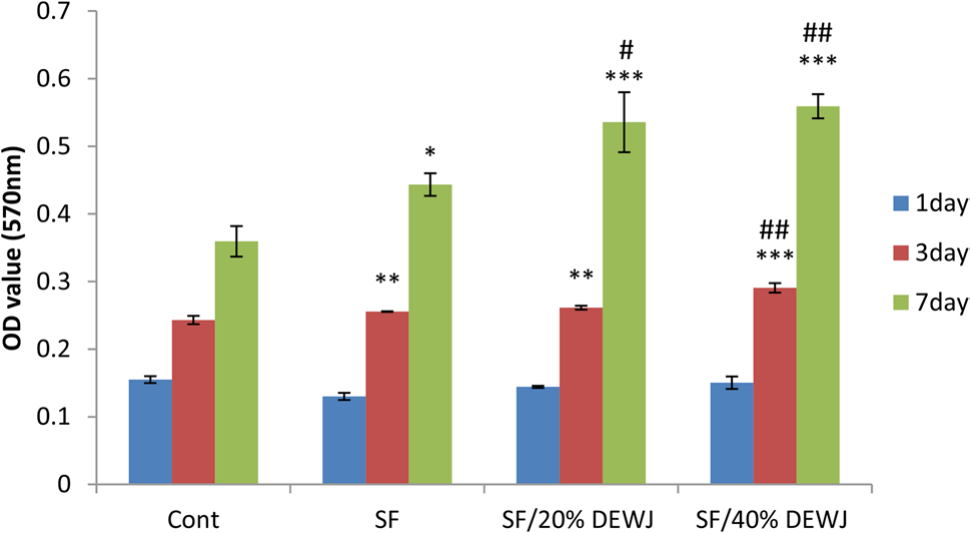
MTT results for SF, SF/20% DEWJ, SF/40% DEWJ hydrogls using hEnSCs. Data are shown as mean ± standard deviation (*n* = 3). * and ^#^ symbols, respectively, indicate comparison with control group and SF hydrogel at same time points (**P < 0.01, ***P < 0.001, ^#^*P* < 0.05, and ^##^*P* < 0.01, *n* = 3). *SF* silk fibroin, *DEWJ* dcellularized extract from Wharton’s jelly, *hEnSCs* human endometrial mesenchymal stem cells

To investigate the potential of SF, SF/20%DEWJ, and SF/40%DEWJ hydrogels as tissue-engineered constructs, their effects on stem cell viability and proliferation were evaluated using MTT assay. The results revealed an incremental pattern for cell population during 7 days of the study in all groups (Fig. 7). The cells influenced by SF/40%DEWJ hydrogel on day 3 and the cells influenced by SF/20%DEWJ and SF/40%DEWJ hydrogels on day 7 indicated a significant increased population than either control or pure SF hydrogel.

## Discussion

Preparing an appropriate scaffold with good biocompatibility and mechanical properties which mimics the native microenvironment of cells is one of the key elements in cartilage tissue engineering. ECMs obtained from decellularization process are natural biomaterials which contain different bioactive molecules and contribute to biological trends. The main goal of tissue decellularization is to simulate the natural niche and microenvironment for culturing cells and bringing them closer to their best function similar to what they do in the body. WJ has a rich ECM which is similar to those of articular cartilage (Xiao et al. 2017). Therefore, decellularization of this tissue is attractive for cartilage tissue engineering applications. Previous studies have used a wide range of decellularization methods including physical, chemical, and enzymatic approaches or their combination with various advantages and disadvantages (Gilpin and Yang 2017). For example, the chemical treatment, especially with the most common substances named sodium dodecyl sulfate (SDS) and triton x-100, has reduced the levels of some growth factors and has also damaged and denatured some collagen fibers (Brown et al. 2011; Hwang et al. 2017). Therefore, choosing an appropriate procedure with minimal damage to the native ECM components contributes to obtaining a more efficient material. To achieve this goal, in the present study, a mild procedure by centrifuge device was chosen and the quality of decellularization and the composition of the obtained material were evaluated. The results of DAPI staining, DNA quantification, and PCR analysis after centrifugation at three different rounds and times verified that the extracts treated at 5000 rpm/30 min and 10,000 rpm/15 min have been completely decellularized.

For further experiments, the extract obtained from centrifugation at 5000 rpm/30 min was used. To define the biochemical components of DEWJ including total proteins, collagens, TGF-β1, and sGAG, the overnight extract was compared with the immediate extract to study the effect of time on extract components. The concentration of total protein in the immediate extract was more than that of the overnight extract. This can be due to the fact that when WJ is exposed to water, most of the permeable proteins immediately enter the water due to the osmotic pressure. At the same time, an increase in the concentrations of soluble, insoluble, and total collagen over time can be related to the swelling of WJ. This swelling enhances during exposure to water and leads to a pressure that isolates some collagen fibrils from the tissue (Meyer 2012). In the case of TGF-β1 and sGAG, there was a minimal increase in concentration over time. It was hypothesized that some TGF-β1 molecules were permeable proteins which enter the water immediately as described before. However, taking the extractions of the rest bound to ECM by disulfide bonds is difficult (Małkowski et al. 2008). Concerning the sGAG content of extracts, the loss of different cell types during decellularization process may account for the reduced amount of sGAG. However, there is still some sGAG content in the DEWJ.

It is known that there is a broad range of interactions between MSCs and their environment which means that the niche or microenvironment of these cells is vital for supporting and stimulating them to do their functions well (Khodadi et al. 2016; Sobolewski et al. 2005). Previous studies have shown the considerable effects of WJ-MSCs conditioned medium on up-regulation of several factors which assist in wound healing and increasing human skin fibroblasts expansion and migration in vivo (Arno et al. 2014). Moreover, replicative senescence of MSCs during cell culture can be efficiently suppressed using human WJ extract (Hao et al. 2013) and some studies have discussed the effect of WJ extract coating on adhesion, proliferation (Dan et al. 2017), and osteogenic differentiation of MSCs (Ahmadi et al. 2017). Also, WJ-ECM has accelerated the differentiation of cells into myofibroblasts and assisted in wound healing (Bakhtyar et al. 2017, Beiki et al. 2017). Xiao et al. (2017) compared WJ-ECM with articular cartilage ECM and reported the potential of WJ-ECM scaffold for future cartilage tissue engineering applications. Therefore, the DEWJ obtained in this study is expected to have beneficial effects on cartilage tissue-engineered constructs. To assess this hypothesis, after evaluation of two extracts of WJ, we chose the overnight extract as a base material for investigating how its different concentrations can influence the hEnSC viability and proliferation.

To prepare a cartilage tissue-engineered construct to carry DEWJ and deliver it to the cells, SF was chosen because it is a typical biomaterial with a broad range of applications including drug delivery and scaffold synthesis in various forms by different methods especially in the case of hydrogels (Kapoor and Kundu 2016). Also, it has some attractive features such as low immunogenicity, low inflammatory response, suitable biocompatibility, appropriate biodegradability, and unique biochemical and mechanical properties (Bhardwaj et al. 2011). In a previous report, a lyophilized SF hydrogel was introduced as a good delivery matrix for antibiotics because of its merits: (1) easier approach due to making and storing therapeutic protein and delivery matrix simultaneously as well as convenient handling and implantation, (2) removing the effect of shear and temperature stresses on loaded agents, (3) maintaining protein stability after entrapment process for a long time (Guziewicz et al. 2011). In addition, Chao et al. (2010) have also reported physical crosslinking of SF by sonication to fabricate a hydrogel for cartilage tissue engineering applications. Thus, in the present study, SF was used to produce a cartilage tissue-engineered construct in the form of a hydrogel and it was attempted to maintain its unique properties and to add the benefits of DEWJ.

FTIR analysis indicated that the biochemical composition of SF has not been changed and blending between SF and DEWJ has probably been physical because no new peaks were seen in hybrid hydrogels. Rheological characterization of hydrogels including amplitude sweep and frequency sweep verified the gel-like or solid viscoelastic behavior of hydrogels because of the higher values of *G*′ compared with *G*″. In the amplitude sweep test, hybrid hydrogels predominantly displayed gel-like properties under the influence of a two-fold amplitude strain before intersection point compared with pure SF hydrogel. In other words, they can maintain their gel-like behavior in response to more strain percentages and, therefore, they have a stronger structure than pure SF hydrogel. Also, *G*′ and *G*′′ values were about 3.5 and 7 times higher for SF/40%DEWJ and SF/20%DEWJ hybrid hydrogels, respectively, in comparison with SF hydrogel which means a more stable system. In the frequency sweep test, the *G*′ and *G*″ values increased relatively identically for all hydrogels which means that the viscoelastic behavior is solid-like and depends on the frequency (Le Goff et al. 2015). These results are in agreement with those of Singh et al. (2016) who discussed agarose/SF blended hydrogels. The morphology of different hydrogels were approximately similar. Although the pore size of hybrid hydrogels was significantly more than that of pure SF. The morphology of the cells and their attachment on the hydrogels were studied after 48 h of culture using FESEM.

The findings of MTT assay with regard to different concentrations of DEWJ in culture media demonstrated an increased pattern in the viability and proliferation of hEnSC for all concentrations from day 1 to day 7. The first significant increase was observed for 20, 30, and 40% concentrations on day 3. However, at 40% concentration, a significant and continuous increase was observed during the whole time period of the study. To assess cell viability and proliferation of hEnSCs influenced by different hydrogels, MTT assay was done on days 1, 3, and 7. For all kinds of hydrogels, it was observed that cell viability and proliferation dramatically increased with the passage of time from day 1 to day 7 (*p* < 0.05). This obviously showed the biocompatibility of the fabricated hydrogels. In addition, the hybrid hydrogels showed more cell viability and proliferation compared with both control and pure SF hydrogel after 7 days. However, there was no significant difference between SF/20%DEWJ and SF/40%DEWJ hybrid hydrogels on the two mentioned time periods. It is possible that some ingredients of DEWJ such as growth factors are released from SF-based hydrogels and enhance cell viability and proliferation. This fact can confirm that DEWJ maintains its biological function even after mixing with SF under manufacturing factors.

## Conclusions

This work explains the fabrication and characterization of a new construct made of DEWJ and SF to combine their attractive features intended for cartilage tissue engineering. In this construct, DEWJ was used due to its similarity with cartilage extracellular matrix and SF was chosen as the base material for scaffolding due to suitable mechanical properties and easy processability. The results showed that DEWJ in hybrid hydrogels can enhance mechanical properties compared with pure SF hydrogels. Furthermore, DEWJ at 20% or 40% concentrations in SF-based hydrogels can improve cell viability. Therefore, SF/DEWJ hybrid hydrogels possibly have the potential for future cartilage regeneration.
